# Influence of natural variations in eating rate and bolus properties on postprandial glucose and insulin responses in healthy adults

**DOI:** 10.1007/s00394-026-04051-2

**Published:** 2026-07-03

**Authors:** Zhen Liu, Marieke van Bruinessen, Lise A. J. Heuven, Marlou P. Lasschuijt, Markus Stieger, Ciarán G. Forde

**Affiliations:** Sensory Science and Eating Behavior Group, Division of Human Nutrition and Health, Wageningen University and Research, Wageningen, the Netherlands

**Keywords:** Eating rate, Oral processing behaviour, Postprandial glucose, Insulin and glucagon, Bolus properties

## Abstract

**Purpose:**

We investigated to what extent natural variations in test-meal oral processing, eating rate and bolus properties influence postprandial glucose responses, while also considering the role of insulin and glucagon.

**Methods:**

Healthy participants (*n* = 33, age: 27.3 ± 5.7 y, BMI: 23.7 ± 2.0 kg/ m^2^) consumed a mixed meal with a fixed carbohydrate load while being video-recorded to assess their eating rate (ER) and oral processing behaviours. Bolus samples at the point of swallowing were collected to determine saliva uptake and particle characteristics. Blood samples were collected before and over a 3-h postprandial period to measure glucose and plasma insulin, glucagon, C-peptide concentrations. Participants were grouped into Slower- or Faster-eaters based on their ER for the test meal.

**Results:**

Slower-eaters (*n* = 17) consumed their meal 53% slower than Faster-eaters (*n* = 16)(*p =* 0.001), with 1.4 times more chews/g and 91.2% longer oro-sensory exposure time (OSE time). Slower- and Faster-eaters did not differ in their total postprandial glucose (PPG) responses. Early (0–30 min) and total (0–180 min) glucose iAUC values were higher in Slower-eaters (20.21% and 55.27%, respectively), but not reaching statistical significance. In contrast, Slower-eaters showed significantly higher insulin and C-peptide responses. Higher total insulin iAUC was associated with higher OSE time (*R* = 0.41) and more chews/bite (*R* = 0.44). Despite differences in bolus properties between Slower- and Faster-eaters, no significant associations were found between bolus properties and PPG or insulin responses.

**Conclusion:**

Enhanced chewing and prolonged oro-sensory exposure during slower eating may stimulate postprandial insulin release, contributing to modulating glucose homeostasis.

**Supplementary Information:**

The online version contains supplementary material available at 10.1007/s00394-026-04051-2.

## Introduction

With the rising global prevalence of diabetes, increasing attention has been directed toward understanding the influence of eating behaviours on blood glucose regulation [1, 2]. While changes in blood glucose levels are influenced by the composition and glycaemic load of a meal, research has shown wide variations in individual postprandial responses to the same fixed carbohydrate load consumed within a test meal [3, 4]. One potential factor contributing to this inter-individual variability may be natural variation in oral processing behaviour among individuals in either the rate of eating or properties of the food bolus at swallow. Recent studies suggest that the way people eat, chew and orally-process food in the mouth can significantly influence digestion and subsequent blood glucose responses [5]. However, previous research has relied on instructions to participants to change their eating speed and it remains unclear to what extent natural variations in eating rate and oral processing behaviour can influence postprandial glucose and insulin response among healthy participants.

Oral processing behaviour describes the micro-structural patterns of eating that include average bite size, chews *per* bite and time spent chewing (oro-sensory exposure time, seconds), and can influence bolus quality at swallow by changing bolus particle size, surface area and saliva uptake [6, 7]. This phase of digestion plays a critical role in determining the rate and extent of nutrient digestion and kinetics of postprandial glucose and insulin responses [8, 9]. Previous research has shown that increasing the masticatory cycles leads to smaller bolus particle size, greater bolus surface area, and greater saliva incorporation, which can alter temporal glucose responses to a fixed carbohydrate load. Supporting this mechanism, in vitro studies using rice have demonstrated an inverse correlation between bolus particle size and the extent of starch hydrolysis during gastrointestinal digestion [10, 11]. Additionally, extended chewing promotes starch hydrolysis in the oral cavity due to a longer period of interaction between a larger bolus surface area and increased secretion and uptake of salivary amylase. This early enzymatic activity may contribute to higher glucose levels, as starch digestion begins in the mouth and continues until enzyme deactivation with low gastric pH [12]. Together, these findings suggest that individual differences in mastication behaviour may contribute to variability in starch digestion and glucose responses [13].

Insulin and glucagon are two key hormones that help to regulate glucose homeostasis [14]. Insulin is released from beta-cells of the pancreas following a meal in response to rising blood glucose levels. It helps glucose uptake in muscles, liver, and fat tissue, lowering blood sugar levels. Glucagon is secreted in response to low-levels of blood glucose, to stimulate the release of stored glucose to support brain functioning [15]. Research investigating the effects of individual eating rates in participants at higher risk for type 2 diabetes suggests that slowing their eating enhances early glucose release which stimulates greater early insulin responses that help support euglycemia [16]. Beyond these nutrient-driven mechanisms, insulin is released during the cephalic or pre-digestive phase, before glucose absorption with these responses triggered by sensory cues such as the sight, smell or taste of food [17]. For instance, insulin release has been observed within 10 min of meal onset, independent of increases in blood glucose levels. Prolonged oro-sensory exposure time has also been associated with greater insulin release [18], and increased chewing may enhance the secretion of gut hormones, such as GLP-1, which further promotes insulin secretion [2, 19].

Despite growing evidence linking oral processing behaviour to post-prandial metabolic outcomes, most existing studies have relied on experimental manipulations of behaviour, such as instructing participants to chew a set number of times *per* mouthful, or placing restrictions to eat more slowly. While informative, such interventions may not accurately reflect natural variations in individuals’ eating behaviours and no studies have linked these behaviours to bolus properties at swallow and the subsequent glucose and neuro-endocrine response. Moreover, the reproducibility and reliability of these effects on glycaemic control and hormone regulation remain to be firmly established [20]. Given the observed variability in natural oral processing behaviours and individual differences in metabolic responses to the same nutrient load, we sought to investigate how individual differences in oral processing behaviour influence bolus properties at swallow and subsequently influence postprandial glucose response, and how this is mediated through insulin and glucagon release or inhibition.

In the current study, we investigated the influence of natural differences in oral processing behaviour on postprandial glycaemic responses to a fixed-portion meal. Eating rate, a key integrative measure that reflects the combined effects of bite size, chewing frequency, and oral exposure time, was considered the primary variable of interest to stratify our participants. We hypothesized that a slower eating rate would be characterized by a higher number of chews *per* bite, and longer oro-sensory exposure duration, and that this would result in early glucose release through a combination greater bolus surface area, time in mouth and greater saliva uptake, we further hypothesized that slower eating would lead to increased insulin and decreased glucagon secretion in response to early glucose release. Finally, we explored whether individual variability in glucose, insulin and glucagon responses could be explained by differences in oral processing behaviour and bolus properties at swallow between Slower- and Faster-eaters.

## Materials and methods

### Participants

Participants were recruited from Wageningen and the surroundings via social media, flyers, and mailing lists. Participants were eligible to participate if they were aged between 21 and 50 years and had a BMI between 21 and 27 kg/m^2^, with self-reported general health, normal appetite, and without self-reported eating difficulties. Participants were excluded if they had allergies or intolerances to the test foods, were smokers, or used any medication that affected study outcomes.

Prospective participants were invited for a screening session to measure blood pressure (excluded if below 90 and/or below 60 mm hg), fasted glucose level (inclusion range: 3.5-8.0 mmol/L), and haemoglobin (Hb) values (inclusion range: 7.5–11.0 mmol/L female and 8.5–11.0 mmol/L male). Participants were further profiled for their stimulated salivary flow and salivary α-amylase activity [16]. Gut transit time was assessed twice during the study period under participant’s habitual dietary conditions using a blue dye method [21].

### Study procedure

Postprandial glucose and pancreatic hormone responses were assessed after consumption of a fixed-portion meal under participants’ habitual eating rate and oral processing behaviour. All participants completed repeated tests on three occasions within a 7-week period. On each occasion, participants came to the research centre after an overnight fast, followed by consumption of the mixed meal tolerance test (MMTT). The meal consisted of a fixed portion of a rice-based porridge (202 g) and 200 mL of chocolate milk (402 g, 552 kcal, 77 g CHO, 34 g mono- and disaccharides, 15 g fat, 25 g protein). Participants were instructed to consume the chocolate milk within 1 min to standardize the protocol across participants, after which they consumed the rice-based porridge as they normally do. All meals were consumed within approximately 10 min, with one meal completed in 12.5 min. The entire meal was video-recorded by a webcam (Logitech HD c310), which they could not see, to assess ER (g/min) and oral processing behaviours post-hoc. Eating rate was based on rice-based porridge only, as it reflects mastication-related oral processing. Before the meal, participants were cannulated and blood samples were drawn at t = − 10 and t = − 5 (baseline samples) and at t = 10 (end of the meal), 15, 30, 45, 60, 90, 120 and 180 min to measure changes in blood glucose, insulin, C-peptide, and glucagon levels. Following the final blood draw, participants were provided with an extra 10 g of the porridge for bolus collection. Each participant completed a total of 3 MMTT sessions over a 7-week period.

The current study is a secondary analysis of a randomized controlled trial approved by the Medical Ethical Committee ‘East-Netherlands’, The Netherlands (ABR: NL83462.091.23) and complied with the Declaration of Helsinki for Medical Research involving Human Subjects. The trial was pre-registered at clinicaltrials.gov (NCT06113146), and the study design was described in the protocol [22]. Main outcomes of the trial are published elsewhere [23]. Written informed consent was obtained from all participants.

The trial was powered based on the primary outcome. We conducted an additional post-hoc power calculation (G*Power, Windows version 3.1.9.7) for this secondary analysis. With the available sample size, the study had 80% power at a two-sided significance level of 5% to detect a between-group difference in glucose iAUC corresponding to a large effect size (Cohen’s d = 1.1).

### MMTT meal preparation

Rice-based porridge was freshly prepared on the morning of each test day. 500 g of microwave rice (Lassie Toverrijst 1-minuutje, Lassie, the Netherlands) was heated in a microwave for 1.5 min and allowed to cool. The rice was then mixed with 385 g of rice pudding (AH Rijstdessert, Albert Heijn, The Netherlands), 75 g of whey protein powder (Impact Whey Protein, MyProtein, UK), and 27 g of cooking fat (AH Bak & Braad, Albert Heijn, The Netherlands). The mixture was whisked until homogeneous and portioned into 202 g servings. The meal consisted of 202 g of a rice-based porridge and 200 mL of chocolate milk. The total meal weighed 402 g and provided 552 kcal, 77 g of carbohydrates (of which 34 g were mono- and disaccharides), 15 g of fat, and 25 g of protein. Detailed nutritional information for each ingredient is provided in the Supplementary Table S1.

### Bolus collection

Participants were provided with additional rice-porridge samples (10 g) to assess individual oral processing responses to a standardized portion. Participants were asked to chew each sample in their normal way to the point of swallowing and then spit out the bolus. The standardized 10 g portion was selected based on previous described method [24] to minimize variability arising from differences in bite size between participants and allow oral food breakdown to be compared across participants under controlled conditions. Participants completed the procedure in duplicate and were provided with water to rinse their mouths between bites. Expectorated boli were stored immediately on ice and analysed within 10 min. Aliquots of expectorated boli were split for analysis of bolus saliva uptake (3 g) and bolus particle properties (1 g).

### Determination of bolus saliva uptake

Saliva uptake of each bolus sample was determined by analysing the dry matter content [11]. Bolus samples and test meal samples were placed on pre-weighted aluminium dishes and dried at 105 °C until constant weight. Saliva uptake (%) was calculated from the difference in water content between bolus and meal samples.

### Characterization of bolus particle properties

Bolus (0.5–1.0 g) was placed in a Petri dish (120 × 120 × 17 mm) and weighed. Bolus particles were gently separated using a spatula. After the samples were scanned (Canon CanoScan 9000 F Mark II), bolus particle properties (average particle size, number of particles/g, total surface area/g) were determined using image analysis [24]. To reduce noise, particles smaller than 0.07 mm^2^ or with a circularity < 0.15 were discarded from data analysis.

### Oral processing behavioural coding of MMTT meals

Post-hoc behavioural coding was completed on video-recordings of each participant consuming the MMTT using ELAN software (ELAN 5.8, Max Planck Institute for Psycholinguistics, The Language Archive, Nijmegen, Netherlands), using a coding scheme developed previously that provides objective and quantitative measures of a series of pre-defined oral processing behaviours [25]. The coded behaviours included four point events (bite, chew, swallow, sips) and three continuous events (duration of each bite, duration of each sip, and total oral exposure time). The videos from test sessions were then coded separately by the two trained coders, after which all coding was cross-checked between the coders. A subset of meal video recordings was re-coded independently in line with a previously published method [25], and intraclass correlation coefficients (ICC) were between 0.97 and 1.00 across all coded behaviours (Supplementary Table S2), indicating excellent consistency [26]. From coded behaviours, a series of oral processing measures were derived, including ER (g/min), bite size (g), chews/bite (n), chews/g (n), and chewing frequency (n/s) based on consumption of the rice-based porridge, as mastication-related oral processing behaviours are not directly comparable for liquids. Oro-sensory exposure time (OSE time, in seconds) was based on the entire meal.

### Postprandial glucose and pancreatic hormone responses

Blood glucose levels were measured directly after sampling with a blood glucose meter (FreeStyle Freedom Lite) using a blood sample collected via the intravenous cannula. Blood samples for insulin, C-peptide and glucagon were collected in 3-mL EDTA-coated tubes containing aprotinin (from bovine lung inhibitors; Hoffmann-La Roche AG ) and DPP-IV inhibitor (Merck) to prevent endocrine degradation. Blood samples were then centrifuged at 1200 x g at 4˚C for 10 min. Plasma was divided into aliquots and stored at -80 ˚C for later insulin, C-peptide, and glucagon analysis using a multiplex assay (Meso Scale Discovery, Rockville, MD, USA). Assay performance characteristics, including detection ranges, intra- and inter-assay coefficients of variation (CVs), are summarized in the Supplementary Table S3.

### Data analysis

All variables were tested for normality statistically using the Shapiro-Wilk test prior to any statistical comparison. Insulin, C-peptide and glucagon concentrations were not normally distributed and thus were log-transformed prior to further analysis. Blood glucose concentration, plasma insulin, C-peptide and glucagon concentrations measured at 10 and 5 min before meals (T=-10 and T=-5) were averaged to define baseline (fasting) levels. Baseline values were included as covariates in the linear mixed-effects models (LMMs) assessing postprandial responses.

Eating rate (g/min) was first included as a continuous variable in LMMs to assess its effect on PPG and postprandial hormone concentrations across time points, with analyses initially performed separately for each of the three MMTT occasions. To evaluate the reproducibility of these associations between ER and postprandial responses across repeated MMTT occasions, an additional interaction term between eating rate, time point, and test occasions (ER*Timepoint*Test) was included. As the associations were consistent across test occasions, final models included eating rate, time point and their interaction (ER*Timepoint), with participants included as a random effect accounting for repeated measures.

Participants were then stratified into groups to facilitate visualization of the postprandial responses and to provide a more intuitive illustration of the magnitude and temporal pattern of the observed differences between participants with relatively faster and slower eating rates. Eating rate, our primary variable of interest, showed excellent consistency (ICC: 0.956 (0.923; 0.977)) across the three MMTT occasions and was used to group participants. Participants were classified into Slower- or Faster-eaters using a median split based on the average ER across three replicate test meals. Grouping classifications based on the average ER showed > 95% agreement with classifications obtained from each test meal separately and showed broad agreement with alternative classification approaches, including hierarchical clustering. LMMs with subject included as a random effect to account for repeated measures were used to analyse group differences in oral processing behaviours, bolus properties, fasting status (glucose, insulin, C-peptide, glucagon), and iAUC values (glucose, insulin, C-peptide, glucagon). To examine the effects of ER-group, time point, and their interaction on postprandial concentrations of glucose and hormones, repeated-measures LMMs were conducted. Finally, Spearman partial correlations were performed across all participants (*N* = 33) to explore associations between oral processing behaviours, bolus properties, and iAUC values of glucose and hormones, controlling for participant dependency. Given the association between gender and eating rate, as well as the potential influence of gender on postprandial responses and gut transit time, gender was included as a covariate in all models. Accordingly, all reported associations with eating rate as a continuous variable and differences between the Faster- and Slower-eater groups were adjusted for gender.

All statistical analyses were performed using R software (version 4.4.3). Two-tailed *p* values < 0.05 were considered statistically significant. Analysis on the main outcomes was performed using LMMs using the package ‘lme4’, ‘lmerTest’. Spearman partial correlation was conducted using the ‘ppcor’ package.

## Results

### Participant characteristics

Participants (*N* = 33, M = 17) characteristics and their assigned ER group are summarised in Table 1, with a mean (± SD) age of 27.3 (5.7) years, a BMI of 23.7 (2.0) kg/m^2^ and fasting glucose within the normal range. Participant characteristics were largely comparable between groups after stratification, except for gender distribution. Slower-eaters (*n* = 17) had an average of 13.2 h longer gut transit time compared with Faster-eaters (*n* = 16). No differences in stimulated saliva amylose activity, saliva flow, fasting glucose, insulin, glucagon, and C-peptide levels between groups (Table 2).

**Table 1:** Participant characteristics. Data shown as the mean (SD)

	All (*n* = 33)	Group	
		Faster-eaters (*n* = 16, M = 11)	Slower-eaters (*n* = 17, M = 6)
Age (years)	27.3 (5.7)	26.4 (4.4)	28.1 (6.6)
Weight (kg)	70.7 (10.1)	76.1 (11.1)	66.2 (6.5)
Height (cm)	173.0 (9.8)	177.4 (10.9)	169.3 (7.3)
BMI (kg/m^2^)	23.7 (2.0)	24.3 (2.0)	23.1 (1.9)
Saliva amylase activity (U/mL)	121.9 (45.7)	118.5 (46.3)	125.5 (46.0)
Saliva flow rate (g/min)	1.32 (0.71)	1.23 (0.65)	1.41 (0.76)
Gut transit time (h)	29.1 (15.7)	22.1 (12.0)	35.3 (16.2)

**Table 2:** Fasting glucose and hormone levels of Slower- and Faster-eaters. Fasting glucose levels are shown as mean (95% confidence intervals), other hormones are shown as geometric means (95% confidence intervals). Data from each participant were collected across three test sessions. Statistical comparisons between groups were performed using a LMM, with participants included as a random effect. Reported *p*-values correspond to the main effect of ER group

Fasting parameter	Faster-eaters (*n* = 16, M = 11)	Slower-eaters (*n* = 17, M = 6)	*p*-value
Glucose (mmol/L)	4.42 (4.19, 4.65)	4.33 (4.12, 4.54)	0.135
Insulin (µU/mL)	12.31 (10.70, 14.17)	11.43 (10.04, 13.02)	0.595
Glucagon (pmol/L)	10.66 (9.30, 12.22)	10.76 (9.49, 12.19)	0.778
C-peptide (ng/mL)	1.57 (1.42, 1.73)	1.50 (1.37, 1.65)	0.938

### Oral processing behaviour and bolus properties

Oral processing behaviours and bolus properties were highly consistent within-participant across all three MMTT occasions (Supplementary Table S4). Intraclass correlation coefficients (ICCs) ranged from 0.84 to 0.96 for oral processing behaviours, except for the number of total bites, reflecting good to excellent reliability. Bolus particle size showed good consistency (ICCs: 0.760 to 0.831). Reported values for oral processing behaviour and bolus properties represent the mean of three replicate tests.

Compared with Faster-eaters (mean ER: 64.55 ± 7.50 g/min, SE), Slower-eaters (mean ER: 30.06 ± 1.21 g/min, SE) had an average 53% lower ER for the test meal (*p* = 0.001), consumed on average 2.5 g less food *per* bite (*p* = 0.028), and had 15 more chew cycles *per* bite (*p <* 0.001, Table 3). For each g of food, Slower-eaters chewed on average 1.4/g (*p* < 0.001) times more than Faster-eaters. Consequently, compared to Faster-eaters, Slower-eaters had a 91.3% longer oro-sensory exposure time (OSE time, *p* < 0.001). No difference was found in chewing frequency (chews/second).

**Table 3:** Oral processing behaviour (Bite size, Chews/bite, Chews/g, Chewing frequency, OSE time, Eating rate) and bolus property (Number of particles, Total surface area, Average size, Saliva uptake) differences between Slower- and Faster-eaters. Data shown as mean (SE) and statistically compared between groups using LMM with participants as a random factor. Reported *p*-values correspond to the main effect of the ER group

	Faster-eaters (*n* = 16, M = 11)	Slower-eaters (*n* = 17, M = 6)	*p*-value
**Oral processing behaviour**			
Eating rate (g/min)	64.55 (7.50)	30.06 (1.21)	< 0.001
Bite size (g)	14.18 (0.93)	11.86 (0.62)	0.042
Chews/bite (n)	15.16 (1.84)	30.2 (2.99)	< 0.001
Chews/g (n)	1.11 (0.12)	2.51 (0.17)	< 0.001
Chewing frequency (n/s)	1.62 (0.05)	1.67 (0.04)	0.409
OSE time (min)	3.65 (0.25)	6.98 (0.32)	< 0.001
**Bolus properties**			
Number of particles (n/g)	391.55 (55.51)	679.96 (140.49)	0.072
Total surface area (cm^2^/g)	8.06 (0.38)	9.54 (0.81)	0.114
Average size (cm^2^)	0.03 (0.004)	0.02 (0.002)	0.054
Saliva uptake (%)	6.9 (0.61)	6.99 (0.63)	0.924

In response to a fixed portion, Slower-eaters produced 73.66% greater number of particles (*p* = 0.072) and 33.33% smaller mean particle size (*p* = 0.054) compared to Faster-eaters. No significant differences in saliva uptake were found between the two groups.

### Effect of individual eating rate as a continuous variable on postprandial glycaemic responses

We first tested the effect of ER as a continuous variable on PPG, postprandial insulin, C-peptide, and glucagon responses (Table 4). Eating rate had a significant main effect on PPG (F(1,47) = 4.51, *p* = 0.038) responses, with no evidence of a timepoint*ER interaction (F(8,794) = 1.60, *p* = 0.112). There was a significant timepoint*ER effect on postprandial insulin (F(8,785) = 3.47, *p* = 0.001), C-peptide (F(8,783) = 3.10, *p* = 0.002), and glucagon (F(8,781) = 2.03, *p* = 0.040). Post-hoc analysis revealed that a slower ER was significantly associated with higher early insulin levels at 10, 15, 120, and 180 min, accompanied by higher C-peptide levels at 120 and 180 min. In the meantime, lower glucagon levels were found to be associated with slower eating rates at 90 and 120 min (Supplementary Table S6).

**Table 4:** Effects of eating rate (g/min) as a continuous variable, time point, and their interventions on postprandial glucose and hormone response, derived from LMMs accounting for three repeated MMTT occasions within participants

	Glucose		Insulin		C-peptide		Glucagon	
	F	*p*	F	*p*	F	*p*	F	*p*
Eating rate (g/min)	4.51	0.039	1.33	0.254	1.36	0.250	4.31	0.041
Timepoint (min)	11.60	< 0.001	59.47	< 0.001	67.60	< 0.001	6.43	< 0.001
Eating rate (g/min): Timepoint (min)	1.60	0.112	3.47	0.001	3.10	0.002	2.03	0.040

The effect of ER on postprandial glycaemia responses was consistent across tests. No significant three-way interaction between ER, time point, and test occasion was observed (*p* = 0.789–0.997), indicating reproducible associations across the three MMTT occasions (Supplementary Table S5).

### Postprandial glycaemic response between slower- and faster-eaters

#### Postprandial glucose response

**Fig. 1:**
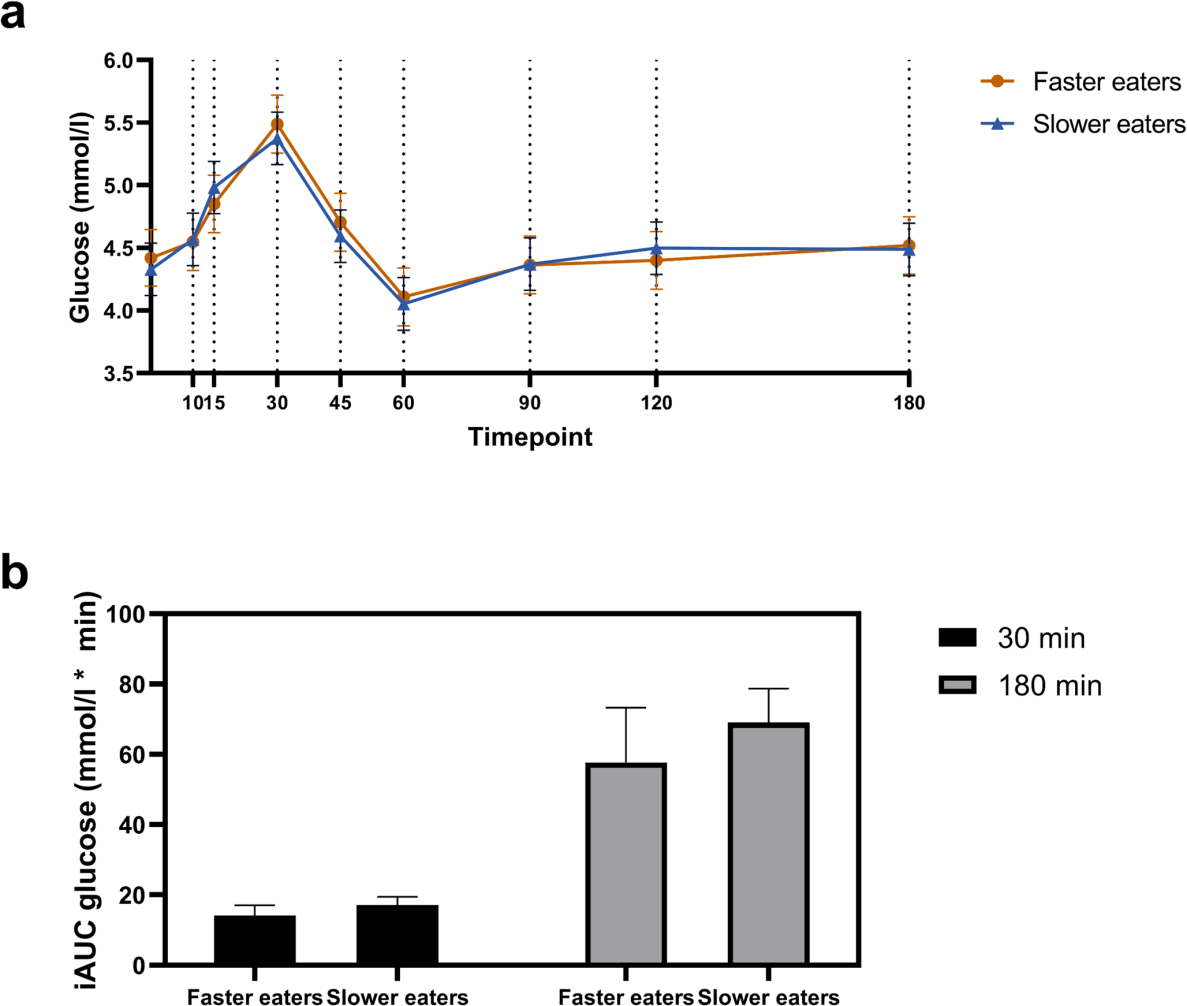
Postprandial glucose response over time (**a**) and incremental area under the curve (iAUC) for 0–30 min and 0–180 min (**b**). Values are shown as means with 95% confidence intervals. No differences in iAUC between groups (LMM, *p* < 0.05)

Figure 1a demonstrates that there was no significant difference in PPG levels between Slower- and Faster-eaters (*p* = 0.888), nor was there a significant interaction between time point and ER groups for PPG levels (*p* = 0.794). Slower-eaters exhibited 20.21% and 55.27% higher early (0–30 min) and total (0–180 min) iAUC glucose values, respectively, compared with Faster-eaters (Fig. 1b). However, these differences were not statistically significant for either early (*p* = 0.228) or total iAUC (*p* = 0.622).

#### Postprandial insulin response

**Fig. 2:**
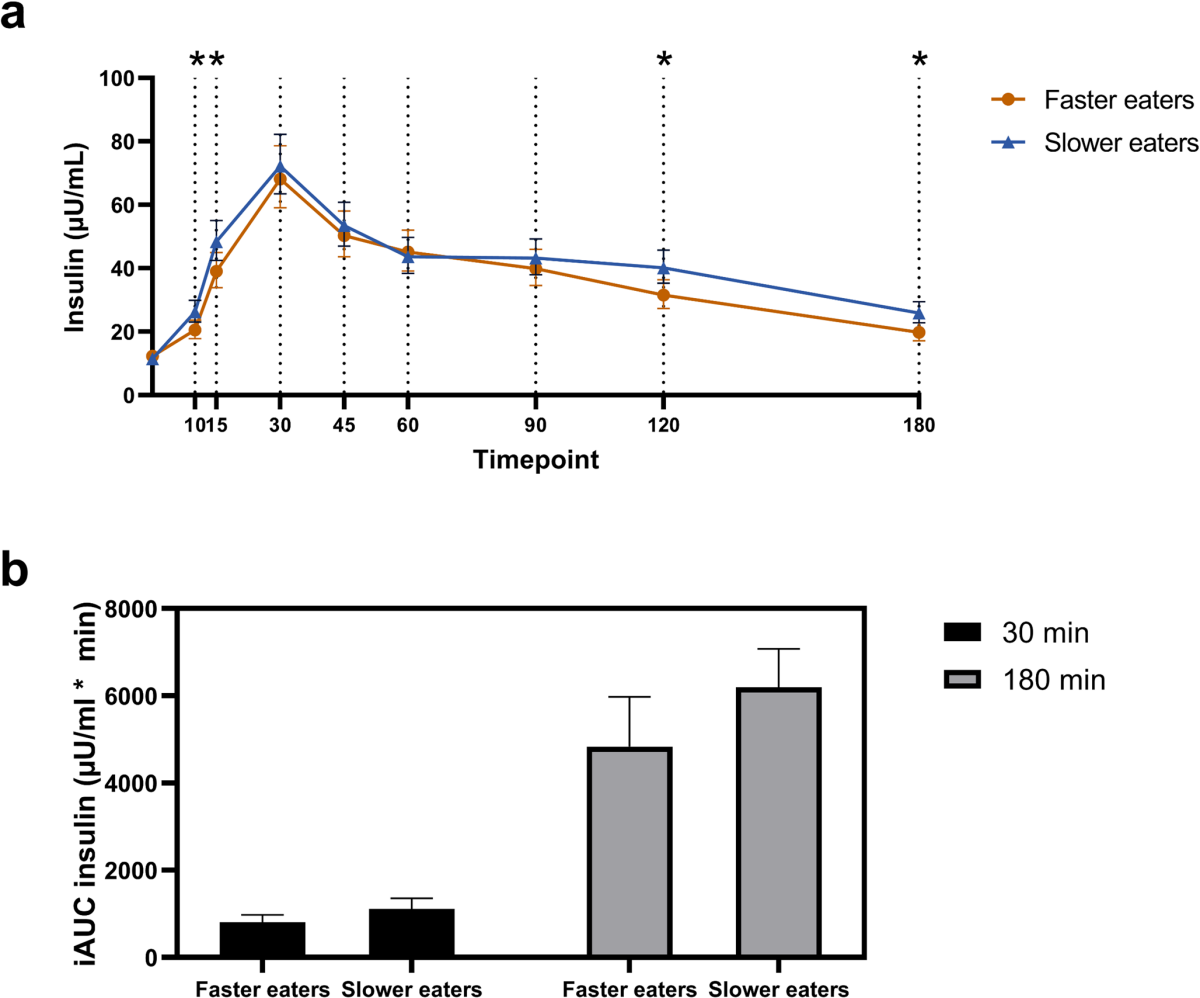
Postprandial insulin response over time (**a**) and incremental area under the curve (iAUC) for 0–30 min and 0–180 min (**b**). Values are shown geometric means with 95% confidence intervals for insulin over time (a), *Significant differences between Slower- and Faster-eaters at certain points (Tukey’s post hoc test, *p* < 0.05). iAUC values are shown as means with 95% confidence intervals (b). No differences in iAUC between groups (LMM, *p* < 0.05)

A significant interaction between timepoint and ER group was observed (*p* = 0.001), indicating differences in the temporal pattern of insulin response between groups (Fig. 2a). Slower-eaters had higher insulin concentrations during the early postprandial phase (mean differences: 5.67 µU/mL at 10 min and 9.35 µU/mL at 15 min) and at later time points (mean differences: 8.62 µU/mL at 120 min and 6.13 µU/mL at 180 min). Consistent with these temporal differences, Slower-eaters had 50.40% higher (*p* = 0.152) early and 56.00% higher (*p* = 0.162) total iAUC insulin than the Faster-eaters (Fig. 2b).

#### Postprandial C-peptide response

**Fig. 3:**
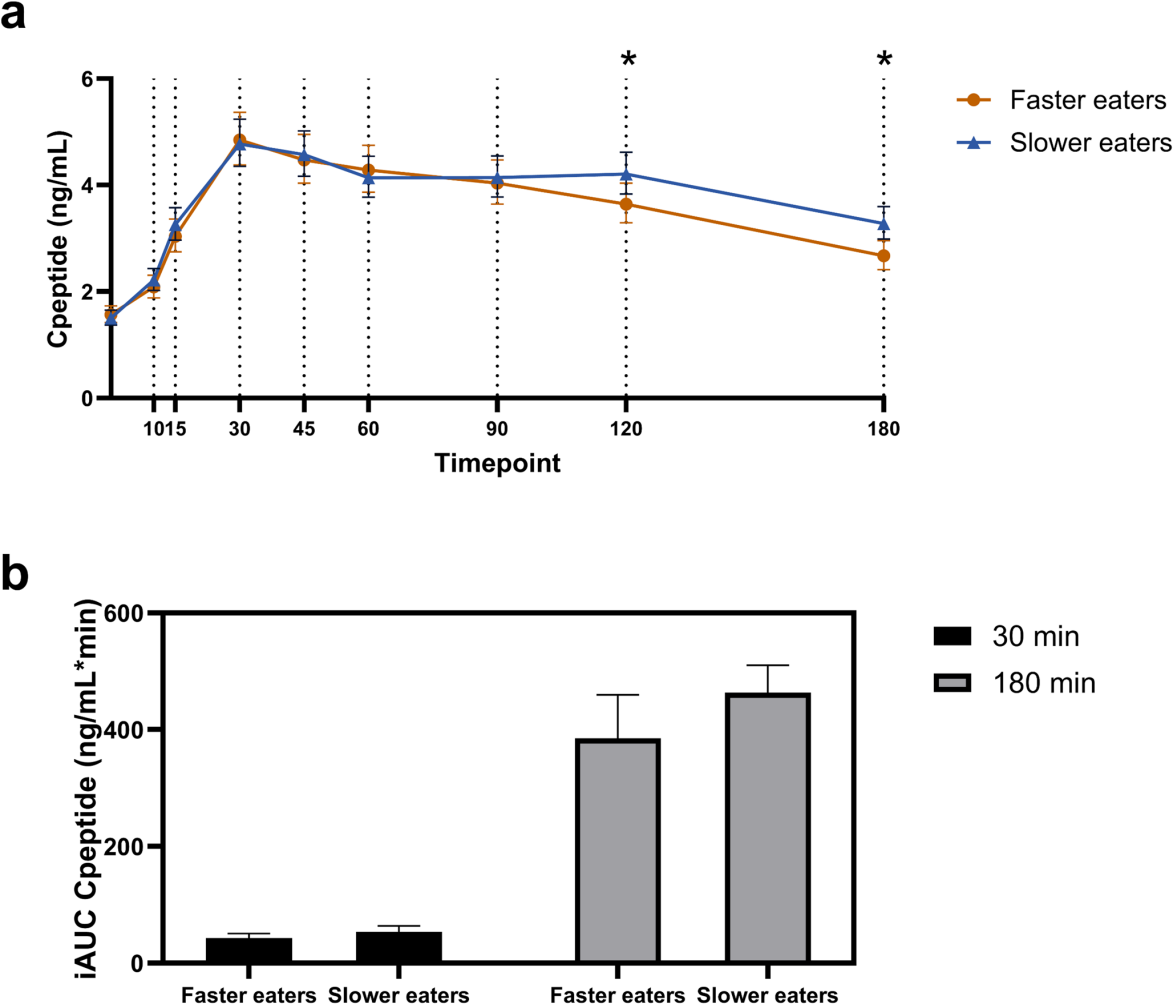
Postprandial C-peptide response over time (**a**) and incremental area under the curve (iAUC) for 0–30 min and 0–180 min (**b**). Values are shown geometric means with 95% confidence intervals for C-peptide over time (**a**), *Significant differences between Slower- and Faster-eaters at certain points (Tukey’s post hoc test, *p* < 0.05). iAUC values are shown as means with 95% confidence intervals (**b**). No differences in iAUC between groups (LMM, *p* < 0.05)

C-peptide responses were also significantly different between ER groups (timepoint*ER group, *p* = 0.002) and followed similar time trends to insulin. At time points 120 and 180 min, significantly higher levels of C-peptide (mean differences: 0.56 ng/mL at 120 min and 0.61 ng/mL at 180 min) were found in Slower-eaters than in Faster-eaters. No significant differences were observed in either early (0–30 min) (*p* = 0.493) or total (0–180 min) iAUC (*p* = 0.480) postprandial periods following the test meal (Fig. 3).

#### Postprandial glucagon response

**Fig. 4:**
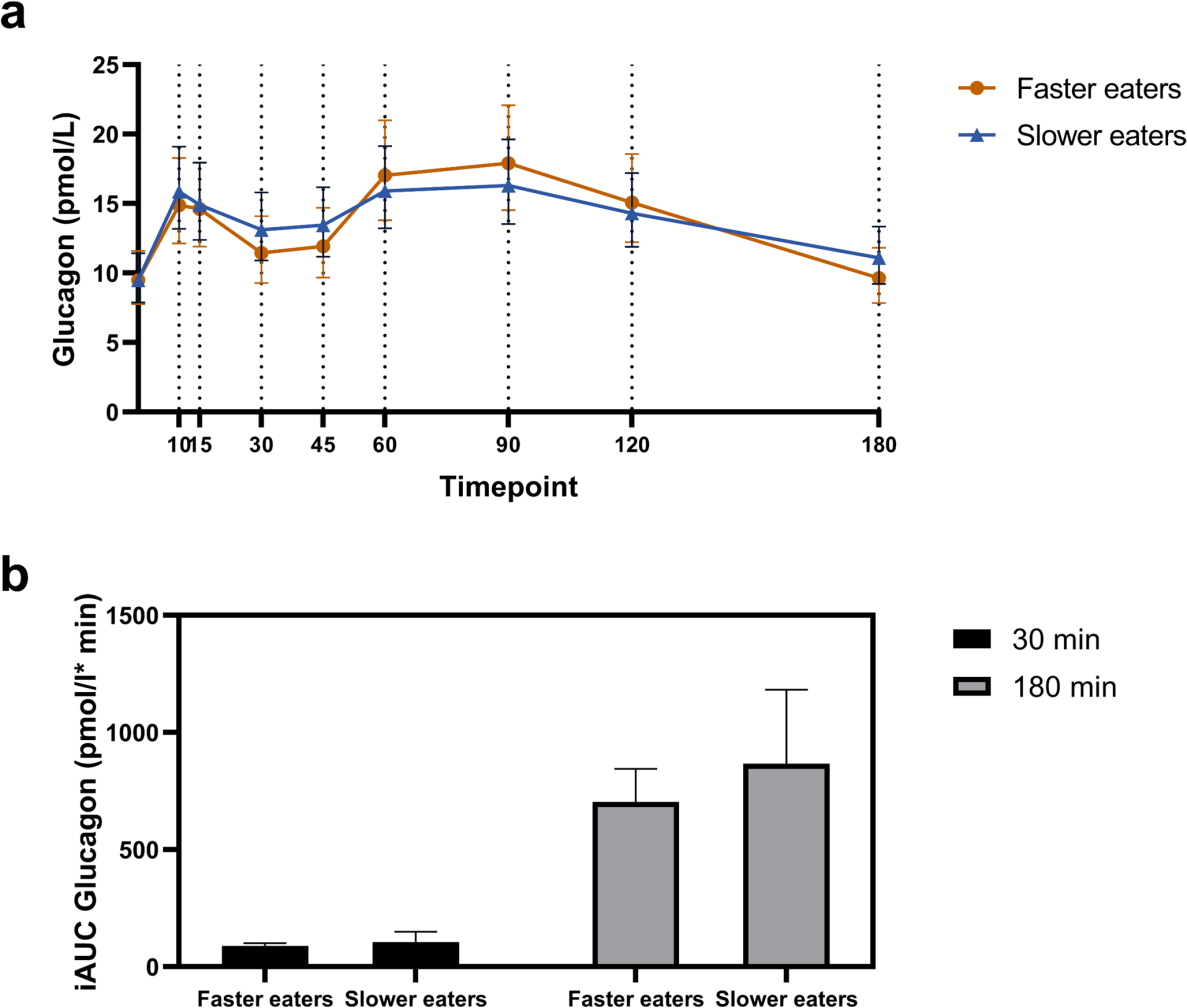
Postprandial glucagon response over time (**a**) and incremental area under the curve (iAUC) for 0–30 min and 0–180 min (**b**). Values are shown geometric means with 95% confidence intervals for glucagon over time (**a**). iAUC values are shown as means with 95% confidence intervals (**b**). No differences in iAUC between groups (LMM, *p* < 0.05)

Postprandial glucagon responses did not differ between ER groups at anytime point and for cumulative measures (iAUC 30 min and iAUC 180 min)(Fig. 4).

### Effect of oral processing behaviour and bolus properties on postprandial glycaemia responses

**Fig. 5:**
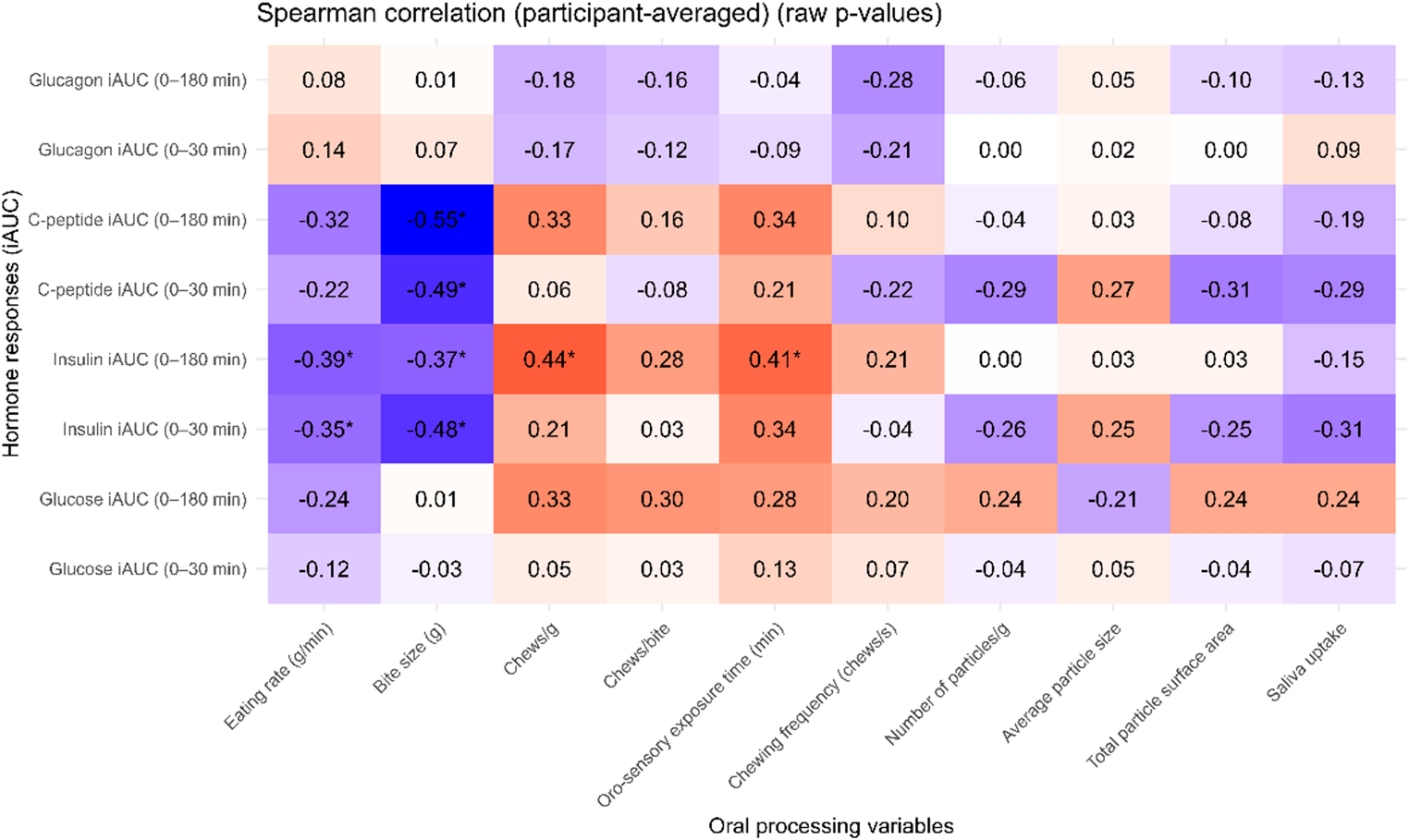
Associations between oral processing variables and postprandial hormonal responses. Heatmap showing correlation coefficients between oral processing variables (x-axis) and postprandial hormonal responses expressed as incremental area under the curve (iAUC) over 0–30 and 0–180 min (y-axis). Color scale indicates the direction and magnitude of correlations (red = positive, blue = negative). Values represent correlation coefficients; *P* < 0.05 is indicated by asterisks

To explore how oral processing behaviour affected glucose response and its postprandial regulations in humans, oral processing behaviour and bolus properties were correlated with PPG, postprandial insulin, C-peptide and glucagon iAUC measures. The analysis revealed significant associations between specific oral processing behaviours, such as ER, chews/g, and OSE time on postprandial insulin and C-peptide iAUC (Fig. 5). Notably, a higher ER was negatively correlated with both early (*R* = -0.38) and total insulin iAUC (*R* = -0.39) and C-peptide iAUC. Similarly, a greater number of chews/g and longer OSE time were positively associated with insulin and C-peptide iAUC. However, total glucose iAUC and glucagon iAUC were not significantly influenced by oral processing behaviour. Effects of bolus properties on PPG response and its regulations remain less effective; only C-peptide iAUC 30 min was found to be significantly influenced by bolus properties, and the results are not as expected.

## Discussion

We investigated the impact of natural variations in eating rate and oral processing behaviour on postprandial glycaemic responses to a fixed-portion MMTT meal. Results demonstrate that differences in individual habitual eating rate and oral processing behaviours are associated with differences in postprandial glucose and insulin dynamics. Though differences did not result in significant differences in total PPG responses, a slower eating rate was associated with a higher insulin concentration in both the early (0–30 min) and later postprandial phase (120–180 min). These effects may be primarily attributable to more chewing and a longer oro-sensory exposure time and early glucose release which stimulated earlier insulin release. Differences in eating rate were reflected in variations in bolus properties, though these were not a significant predictor of the glucose or insulin response. Our findings highlight that an individual’s habitual eating rate plays an important and reproducible role in moderating insulin release and glucose metabolism.

Individuals exhibit considerable variability in their oral processing behaviour, notably in the number of preferred masticatory cycles to form a bolus that is safe to swallow [6]. Our results demonstrate a high degree of individual consistency in eating rate across repeated consumptions of the same meal, representing a stable contribution of oral processing behaviour to variations between individuals in glucose regulation. Slower-eaters were observed to have significantly smaller bite size, higher number of chews/g, and longer OSE time. Faster-eaters did not compensate by increasing chewing frequency, suggesting that their higher eating rate resulted primarily from bigger bites and fewer chews. More extensive mastication increased the total number of particles and total surface area (mm^2^) *per* g of bolus, with a smaller average bolus particle size. However, these differences in bolus particle properties did not reach statistical significance, which might reflect smaller inter-individual variability in boluses compared to eating rate [27]. The use of a standardized 10 g bolus may also have reduced variability attributable to differences in bite size between participants.

The extent of food structure breakdown during mastication can influence enzymatic digestion and nutrient release by changing the duration, available surface area for metabolic activity, and the penetration of digestive enzymes in the bolus. Particles with a smaller size and larger surface areas are more easily broken down by digestive enzymes, which leads to increased glucose release and nutrient absorption [5, 28]. This is supported by previous research, and was a central part of our hypothesis [29–32]. In addition to a smaller bolus particle size, a longer oro-sensory exposure time is also associated with slower eating and may enhance both saliva penetration and early oral starch digestion by extending the time between salivary α-amylase and starch substrates [12]. An extended oral phase could allow greater enzymatic access and partial starch hydrolysis prior to gastric acid inactivation of amylase. A previous study showed that saliva uptake in the bolus plays a role in moderating glycaemic responses in faster and slower eaters, with higher time spent above the normal range for glucose being positively associated with bolus saliva uptake [33]. However, in the present study, saliva flow rate, saliva uptake, and saliva α-amylase activity did not differ between ER groups. Additional analyses examining these variables as continuous predictors did not show meaningful associations with postprandial glucose responses. Potential differences in the present study are more likely due to the oro-sensory exposure time and chews per bite. Although interindividual differences in oral processing behaviour may induce differences in starch hydrolysis, these mechanisms did not lead to significant differences in temporal or total postprandial blood glucose release. Our findings suggest that differences in starch hydrolysis were not the only driver of temporal changes in blood glucose response, and that the additional insulin secretion may have offset differences between the two groups.

This led to our second hypothesis: that variations in oral processing behaviour may influence postprandial hormonal responses, which in turn could affect glucose regulation. Our findings indicate that a slower eating rate is associated with higher early insulin response, potentially related to more extensive chewing and longer oro-sensory exposure time, suggesting that extending chewing may have an incretin effect. Temporal changes in insulin secretion can be influenced by early blood glucose release as well as an accentuated cephalic phase insulin response in response to a prolonged duration of sensory stimulation. In line with this, insulin secretion was found to be higher alongside early increases in blood glucose levels, with significantly higher early insulin levels (0–30 min postprandial) observed in the Slower-eaters group, compared to Faster-eaters. Furthermore, a higher number of chews/g and longer duration of oro-sensory exposure time were both positively correlated with higher insulin iAUC. Previous studies have shown similar results when mastication was enhanced, stimulating a higher anticipatory metabolic response and insulin secretion, that is potentially mediated by increased taste receptor activity [18, 34]. Therefore elevated early insulin levels that are associated with slower eating rates are likely playing an important role in postprandial glucose excursions.

In the current population, slower eating was associated with a longer gut transit time during their habitual diet, and Slower-eaters had on average 13.2 h longer (F(1, 37.7) = 10.87, *p* = 0.002) gut transit time than Faster-eaters. Gastrointestinal transit has been suggested to influence postprandial glucose metabolism, as gastric emptying and small intestinal transit influence the rate of glucose in the duodenum and its subsequent absorption and systemic appearance [35]. A previous study also reported that plasma insulin concentrations at 120 min postprandial were inversely related to gastric emptying rate [36]. A prolonged gut transit time associated with slower eating may therefore lead to a more gradual and prolonged delivery of nutrients through the digestive tract, resulting in sustained nutrient appearance in the circulation [37]. This mechanism may help to explain the higher insulin secretion observed during the later postprandial phase (120–180 min) in Slower-eaters. However, gut transit time in the present study reflects the whole gut transit, including the colonic phase, and represents a habitual characteristic of participants rather than gastric emptying or upper gastrointestinal transit in response to the test meal. Although previous studies have shown that experimentally manipulated slower eating and increased chewing are accompanied by higher postprandial GLP-1 release, a hormone known to reduce gut motility [38], gastric emptying was not directly measured in the present study. Moreover, smaller bolus particle sizes associated with a slower eating rate have also been reported to accelerate gastric emptying [39]. Bolus particle properties may also influence the downstream digestive process, including microbiome fermentation in the large intestine, as reported previously [11]. These observations underscore the complexity of oral processing-related effects on postprandial metabolism, indicating that future studies into the underlying mechanisms.

Glucagon, secreted from pancreatic α-cells, stimulates hepatic glucose production and plays a key role in the regulation of glucose homeostasis, particularly when blood glucose levels begin to decline [40]. In the present study, no significant differences in postprandial glucagon concentrations were observed between Slower- and Faster-eaters. This could be because glucagon secretion is primarily stimulated under conditions of low levels of blood glucose [15]. Notably, glucagon was also secreted during the very early postprandial phase (0–10 min) together with insulin, alongside the initial rise in blood glucose. This coordinated early hormonal response may contribute to maintaining glucose stability across different eating patterns and help explain the absence of significance in postprandial blood glucose levels in the present cohort of healthy participants.

Multiple studies show that experimentally manipulated slower eating and prolonged oro-sensory exposure correlate with elevated insulin secretion [16, 18, 34]. However, findings on postprandial glucose outcomes under the influence of eating rate remain inconsistent across studies and may depend on food properties. For instance, Goh et al. found no difference in postprandial glucose between slow and fast eaters of white rice in healthy males, but observed significantly higher early glucose release among Slower-eaters when consuming the harder rice cake within the same population [5]. These findings suggest that the role of food texture and structural complexity in modifying the metabolic impact of oral processing behaviour. However, postprandial insulin responses were not assessed in the study of Goh et al., limiting the interpretation of the underlying metabolic mechanisms. It is possible that more pronounced differences in glucose responses would be observed for harder, or more structurally complex foods that demand greater masticatory effort and a longer oral processing time. Under such conditions, variations in bolus properties and their subsequent effects on digestion efficiency may lead to a more pronounced effect on the temporal dynamics of postprandial glucose response. This may inform the design of food textures and structures to better manage glucose homeostasis and account for individual differences in preferred oral processing behaviours. In the present study, we utilised a rice-based porridge meal with limited texture, and data were collected among healthy individuals. The total postprandial glucose and insulin responses were not significantly associated with differences in bolus properties, but future research could further explore similar relationships across different meal textures and within a pre-diabetic or clinical population, to evaluate the efficacy of oral processing as a support to maintain euglycemic responses post-meal. Similarly, the current study assessed only acute postprandial responses and does not reflect longer-term daily or weekly variations in glucose excursions.

A limitation of this study is that eating rate was not evenly distributed across sexes, with Faster-eaters being predominantly male. Although analyses were adjusted for sex, residual confounding cannot be fully excluded, and a more balanced sex distribution would strengthen future studies.

Taken together, our findings suggest that more thorough mastication and longer oro-sensory exposure time may primarily influence postprandial glucose regulation through modulation of insulin responses, rather than through differences in bolus-related digestive processes. Our findings suggest that natural variations in eating rate and bolus properties have an impact in moderating the postprandial insulin response, thereby contributing to the regulation of glucose homeostasis. Importantly, these results were consistently observed across three independent test meals within the same population, suggesting these behavioural differences are likely to have a robust impact on glucose regulation over time. Future studies should explore long-term metabolic effects of oral processing behaviour in habitual settings and consider a broader range of food textures and compositions.

## Conclusion

In conclusion, this study provides evidence that natural variations in eating rate and oral processing behaviors can influence postprandial glucose and neuro-endocrine responses, primarily through alterations in insulin dynamics, while postprandial blood glucose levels remain tightly regulated in healthy individuals. Our findings suggest that thorough mastication and longer oro-sensory exposure may modulate postprandial glucose regulation primarily through hormonal mechanisms rather than through differences in bolus properties or digestive efficiency. Results highlight that encouraging a slower eating rate, characterized by increased chewing and prolonged oro-sensory exposure, may have relevance for the postprandial metabolic regulation of blood glucose, and helps to explain observed inter-individual differences in glucose responses to a fixed carbohydrate load.

## Supplementary Information

Below is the link to the electronic supplementary material.

Supplementary Material 1

## Data Availability

The datasets are available from the corresponding author upon reasonable request.

## References

[1] Genitsaridi I, Salpea P, Salim A et al (2026) 11th edition of the IDF Diabetes Atlas: global, regional, and national diabetes prevalence estimates for 2024 and projections for 2050. Lancet Diabetes Endocrinol 14:149–156. 10.1016/S2213-8587(25)00299-2 41412135 10.1016/S2213-8587(25)00299-2

[2] Slyper A, Diabetes (2021) Oral Processing, Satiation and Obesity: Overview and Hypotheses. Metabolic Syndrome Obes 14:3399–3415. 10.2147/DMSO.S314379 10.2147/DMSO.S314379 PMC8323852 34345176

[3] Papakonstantinou E, Oikonomou C, Nychas G, Dimitriadis GD (2022) Effects of Diet, Lifestyle, Chrononutrition and Alternative Dietary Interventions on Postprandial Glycemia and Insulin Resistance. Nutrients 14:823. 10.3390/nu14040823 35215472 10.3390/nu14040823 PMC8878449

[4] Brand-Miller JC, Stockmann K, Atkinson F et al (2009) Glycemic index, postprandial glycemia, and the shape of the curve in healthy subjects: analysis of a database of more than 1000 foods. Am J Clin Nutr 89:97–105. 10.3945/AJCN.2008.26354 19056599 10.3945/ajcn.2008.26354

[5] Goh AT, Chatonidi G, Choy M et al (2021) Impact of individual differences in eating rate on oral processing, bolus properties and post-meal glucose responses. Physiol Behav 238:113495. 10.1016/j.physbeh.2021.113495 34116051 10.1016/j.physbeh.2021.113495

[6] Forde CG, Stieger M (2022) Metabolic Impacts of Food Oral Processing. Oral Processing and Consumer Perception. The Royal Society of Chemistry, pp 137–186

[7] van Eck A, Stieger M (2020) Oral processing behavior, sensory perception and intake of composite foods. Trends Food Sci Technol 106:219–231. 10.1016/j.tifs.2020.10.008

[8] Teo PS, Lim AJY, Goh AT et al (2022) Texture-based differences in eating rate influence energy intake for minimally processed and ultra-processed meals. Am J Clin Nutr 116:244–254. 10.1093/ajcn/nqac068 35285882 10.1093/ajcn/nqac068 PMC9257473

[9] Nadia J, Bronlund J, Singh RP et al (2021) Structural breakdown of starch-based foods during gastric digestion and its link to glycemic response: In vivo and in vitro considerations. Compr Rev Food Sci Food Saf 20:2660–2698. 10.1111/1541-4337.12749 33884751 10.1111/1541-4337.12749

[10] Kim EHJ, Wilson AJ, Motoi L et al (2022) Chewing differences in consumers affect the digestion and colonic fermentation outcomes: in vitro studies. Food Funct 13:9355–9371. 10.1039/D1FO04364A 35972507 10.1039/d1fo04364a

[11] Liu Z, Forde CG, Stieger M, Rubert J (2025) Chewing behavior and bolus particle size of rice influence carbohydrate digestion and gut microbiome metabolism in vitro. Food Chem 492:145404. 10.1016/j.foodchem.2025.145404 40645046 10.1016/j.foodchem.2025.145404

[12] Akturk HK, Rewers A, Joseph H et al (2018) Possible Ways to Improve Postprandial Glucose Control in Type 1 Diabetes. Diabetes Technol Ther 20:S224–S232. 10.1089/dia.2018.0114 29916737 10.1089/dia.2018.0114

[13] Ranawana Viren V, Henry CJK, Pratt Megan M (2010) Degree of habitual mastication seems to contribute to interindividual variations in the glycemic response to rice but not to spaghetti. Nutr Res 30:382–391. 10.1016/J.NUTRES.2010.06.002 10.1016/j.nutres.2010.06.002 20650345

[14] Kumar VM (1999) Neural regulation of glucose homeostasis. Indian J Physiol Pharmacol 43:415–424. 10.1002/J.2040-46032017.TB00749.X;PAGEGROUP:STRING:PUBLICATION 10776456

[15] Claessens M, Calame W, Siemensma AD et al (2009) The effect of different protein hydrolysate/carbohydrate mixtures on postprandial glucagon and insulin responses in healthy subjects. Eur J Clin Nutr 63:48–56. 17851462 10.1038/sj.ejcn.1602896

[16] Goh AT, Choy JYM, Chua XH et al (2021) Increased oral processing and a slower eating rate increase glycaemic, insulin and satiety responses to a mixed meal tolerance test. Eur J Nutr 60:2719–2733. 10.1007/S00394-020-02466-Z/FIGURES/3 33389082 10.1007/s00394-020-02466-z

[17] Smeets PAM, Erkner A, De Graaf C (2010) Cephalic phase responses and appetite. Nutr Rev 68:643–655. 10.1111/J.1753-4887.2010.00334.X 20961295 10.1111/j.1753-4887.2010.00334.x

[18] Lasschuijt M, Mars M, De Graaf C, Smeets PAM (2020) How oro-sensory exposure and eating rate affect satiation and associated endocrine responses—a randomized trial. Am J Clin Nutr 111:1137–1149. 10.1093/AJCN/NQAA067 32320002 10.1093/ajcn/nqaa067 PMC7266691

[19] Xu J, Xiao X, Li Y et al (2015) The effect of gum chewing on blood GLP-1 concentration in fasted, healthy, non-obese men. Endocrine 50:93. 10.1007/S12020-015-0566-1 25758865 10.1007/s12020-015-0566-1 PMC4546692

[20] Suzuki H, Fukushima M, Okamoto S et al (2005) Effects of thorough mastication on postprandial plasma glucose concentrations in nonobese Japanese subjects. Metabolism 54:1593–1599. 10.1016/J.METABOL.2005.06.006 16311091 10.1016/j.metabol.2005.06.006

[21] Asnicar F, Leeming ER, Dimidi E et al (2021) Blue poo: impact of gut transit time on the gut microbiome using a novel marker. Gut 70:1665–1674. 10.1136/gutjnl-2020-323877 33722860 10.1136/gutjnl-2020-323877 PMC8349893

[22] Lasschuijt MP, Heuven LAJ, Marieke, Van Bruinessen et al (2025) Nutrition Bulletin The Effect of Eating Rate of Ultra-Processed Foods on Dietary Intake, Eating Behaviour, Body Composition and Metabolic Responses-Rationale, Design and Outcomes of the Restructure Randomised Controlled Trial. Nutr Bull 0:1–16. 10.1111/nbu.70027 10.1111/nbu.70027 PMC12621179 40926558

[23] Forde CG, Heuven LAJ, van Bruinessen M et al (2025) Eating Rate has Sustained Effects on Energy Intake from Ultra-Processed Diets: A Two-Week Ad Libitum Dietary Randomized Controlled Cross-over Trial. Am J Clin Nutr. 10.1016/j.ajcnut.2025.11.012 41314613 10.1016/j.ajcnut.2025.11.012 PMC13084570

[24] Chen Y, Stieger M, Capuano E et al (2022) Influence of oral processing behaviour and bolus properties of brown rice and chickpeas on in vitro starch digestion and postprandial glycaemic response. Eur J Nutr 61:3961–3974. 10.1007/s00394-022-02935-7 35773354 10.1007/s00394-022-02935-7 PMC9596526

[25] Forde CG, van Kuijk N, Thaler T et al (2013) Oral processing characteristics of solid savoury meal components, and relationship with food composition, sensory attributes and expected satiation. Appetite 60:208–219. 10.1016/j.appet.2012.09.015 23017464 10.1016/j.appet.2012.09.015

[26] Koo TK, Li MY (2016) A Guideline of Selecting and Reporting Intraclass Correlation Coefficients for Reliability Research. J Chiropr Med 15:155–163. 10.1016/j.jcm.2016.02.012 27330520 10.1016/j.jcm.2016.02.012 PMC4913118

[27] Van Der, B I L T A (2011) Assessment of mastication with implications for oral rehabilitation: a review. 10.1111/j.1365-2842.2010.02197.x 10.1111/j.1365-2842.2010.02197.x 21241351

[28] Teo PS, van Dam RM, Forde CG (2020) Combined Impact of a Faster Self-Reported Eating Rate and Higher Dietary Energy Intake Rate on Energy Intake and Adiposity. Nutrients 12:3264. 10.3390/nu12113264 33113792 10.3390/nu12113264 PMC7693136

[29] Goh AT, Chatonidi G, Choy M et al (2021) Impact of individual differences in eating rate on oral processing, bolus properties and post-meal glucose responses. Physiol Behav 238. 10.1016/j.physbeh.2021.113495 10.1016/j.physbeh.2021.113495 34116051

[30] Bornhorst GM, Singh RP (2012) Bolus Formation and Disintegration during Digestion of Food Carbohydrates. Compr Rev Food Sci Food Saf 11:101–118. 10.1111/j.1541-4337.2011.00172.x

[31] Gao J, Lin S, Jin X et al (2019) In vitro digestion of bread: How is it influenced by the bolus characteristics? J Texture Stud 50:257–268. 10.1111/JTXS.12391 30693521 10.1111/jtxs.12391

[32] Gao J, Tan EYN, Low SHL et al (2021) From bolus to digesta: How structural disintegration affects starch hydrolysis during oral-gastro-intestinal digestion of bread. J Food Eng 289:110161. 10.1016/j.jfoodeng.2020.110161

[33] Goh AT, Yao J, Chua XH et al (2023) Associations between oral processing, saliva, and bolus properties on daily glucose excursions amongst people at risk of type-2 diabetes. Food Funct 14:2260–2269. 10.1039/D2FO03060H 36762552 10.1039/d2fo03060h

[34] Zhu Y, Hsu WH, Hollis JH (2013) Increasing the number of masticatory cycles is associated with reduced appetite and altered postprandial plasma concentrations of gut hormones, insulin and glucose. Br J Nutr 110:384–390. 10.1017/S0007114512005053 23181989 10.1017/S0007114512005053

[35] Müller M, Canfora EE, Blaak EE (2018),Gastrointestinal Transit, Time Glucose Homeostasis and Metabolic Health: Modulation by Dietary Fibers. Nutrients 10(3):275. 10.3390/nu10030275 10.3390/nu10030275 PMC5872693 29495569

[36] Horowitz M, Edelbroek MAL, Wishart JM, Straathof JW (1993) Relationship between oral glucose tolerance and gastric emptying in normal healthy subjects. Diabetologia 36:857–862. 10.1007/BF00400362 8405758 10.1007/BF00400362

[37] Holst JJ, Gribble F, Horowitz M, Rayner CK (2016) Roles of the Gut in Glucose Homeostasis. Diabetes Care 39:884–892. 10.2337/dc16-0351 27222546 10.2337/dc16-0351

[38] Gribble FM, Reimann F (2019) Function and mechanisms of enteroendocrine cells and gut hormones in metabolism. Nat Reviews Endocrinol 2019 15(4):226–237. 10.1038/s41574-019-0168-8 10.1038/s41574-019-0168-8 30760847

[39] Zannidi D, Methven L, Woodside JV et al (2025) Variations in oral performance and processing behaviours among older adults: Associations with gastric emptying, postprandial glucose and insulin responses. Exp Gerontol 211:112893. 10.1016/J.EXGER.2025.112893 40957482 10.1016/j.exger.2025.112893

[40] Bagger JI, Knop FK, Holst JJ, Vilsbøll T (2011) Glucagon antagonism as a potential therapeutic target in type 2 diabetes. Diabetes Obes Metab 13:965–971. 10.1111/j.1463-1326.2011.01427.x 21615669 10.1111/j.1463-1326.2011.01427.x

