# Supplementary material for: Influence of natural variations in eating rate and bolus properties on postprandial glucose and insulin responses in healthy adults
Source: Eur J Nutr. 2026 Jul 3;65(5):193. doi: 10.1007/s00394-026-04051-2 (PMC13331850; doi:10.1007/s00394-026-04051-2)
Supplement: Supplementary file 1 — Supplementary Material 1 [file 394_2026_4051_MOESM1_ESM.doc]

**Influence of Natural Variations in Eating Rate and Bolus Properties on Postprandial Glucose and Insulin responses in Healthy adults**

Zhen Liu^1^, Marieke van Bruinessen^1^, Lise A.J. Heuven^1^, Marlou P. Lasschuijt^1^, Markus Stieger^1^, Ciarán G. Forde^1^

^1^Sensory Science and Eating Behavior Group, Division of Human Nutrition and Health, Wageningen University & Research, Wageningen, the Netherlands

List of Supplementary tables and figures:

**Table S1:** Composition and nutritional characteristics of the test meal and its individual ingredients.

| **Individual ingredient** | **Energy (kcal/100g)** | **Total fat (g/100g)** | **Carbohydrate (g/100g)** | **Sugars (g/100g)** | **Fiber (g/100g)** | **Protein (g/100g)** | **Salt (g/100g)** |
| --- | --- | --- | --- | --- | --- | --- | --- |
| Microwave rice | 170 | 1.2 | 35 | 0.2 | 2.2 | 3.7 | 0.4 |
| Rice pudding | 120 | 3 | 20 | 12 | 0.09 | 3.2 | 0.15 |
| Whey protein powder | 381 | 6.3 | 5.9 | 5 | 0 | 75 | 0.46 |
| Cooking oil | 814 | 90 | 0.7 | 0.7 | 0 | 0.4 | 0.03 |
| Chocolatemilk | 87 | 2.7 | 12 | 11.8 | 0 | 3.2 | 0.13 |
| **Per serve** | **Energy**  **(kcal)** | **Total fat (g)** | **Carbohydrate (g)** | **Sugars (g)** | **Fiber**  **(g)** | **Protein (g)** | **Salt**  **(g)** |
| Rice porridge (202g) | 378 | 10 | 53 | 11 | 2 | 18 | 1 |
| Chocolatemilk (200 mL) | 174 | 5 | 24 | 24 | 0 | 6 | 0 |
| Total (402g) | 552 | 15 | 77 | 34 | 2 | 25 | 1 |

**Table S2:** Intraclass correlation coefficients (ICC) between coders with 95% confidence interval for the oral-processing behaviours obtained with video annotation.

| **Oral processing behaviours** | **ICC (95% conference interval)** |
| --- | --- |
| Bites (n) | 0.969 (0.594; 0.999) |
| Chews in total (n) | 0.995 (0.899; 1) |
| Bites duration (s) | 0.999 (0.982; 1) |
| Chewing duration (s) | 0.996 (0.933; 1) |
| Whole meal duration (s) | 0.920 (0.999; 1) |

**Table S3:** Assay performance characteristics of measured hormones.

| **Analyte** | **Detection range** | **Intra-assay CV (%)** | **Inter-assay CV (%)** |
| --- | --- | --- | --- |
| Insulin | 0.32–736 µIU/mL | 2.6 (0.4–6.7) | 12 |
| C-peptide | 14–7,610 pg/mL | 2.3 (0.2–6.5) | 15 |
| Glucagon | 0.13–156 pmol/L | 2.6 (0.1–5.7) | 32 |

**Table S4:** Intraclass correlation coefficients (ICC) between three test occasions with 95% confidence interval for the oral processing behaviours and bolus properties.

|  | **ICC (95% conference interval)** |
| --- | --- |
| **Oral processing behaviours** |  |
| Eating rate (g/min) | 0.956 (0.923; 0.977) |
| Bites (n) | 0.667 (0.485; 0.810) |
| Chews in total (n) | 0.916 (0.853; 0.956) |
| Chews/g (n) | 0.916 (0.853; 0.956) |
| Chews/bite (n) | 0.835 (0.723; 0.911) |
| Chewing frequency (n/s) | 0.902 (0.829; 0.948) |
| OSE time (min) | 0.901 (0.828; 0.948) |
| **Bolus properties** |  |
| Number of particles (n/g) | 0.831 (0.724; 0.906) |
| Total surface area (cm^2^/g) | 0.809 (0.691; 0.893） |
| Average size (cm^2^) | 0.760 (0.620; 0.863） |
| Saliva uptake (%) | 0.648 (0.466; 0.793） |

**Table S5** Consistent effects of eating rate on postprandial glycemia responses across replicate tests (LMMs)

|  | Glucose | | Insulin | | C-peptide | | Glucagon | |
| --- | --- | --- | --- | --- | --- | --- | --- | --- |
|  | *F* | *p* | *F* | *p* | *F* | *p* | *F* | *p* |
| Eating rate (g/min) | 4.43 | 0.041 | 0.91 | 0.346 | 0.734 | 0.396 | 5.14 | 0.027 |
| Timepoint (min) | 11.44 | < 0.001 | 57.71 | < 0.001 | 66.20 | < 0.001 | 6.29 | < 0.001 |
| Eating rate (g/min): Timepoint (min) | 1.57 | 0.130 | 3.33 | 0.001 | 3.08 | 0.002 | 2.14 | 0.030 |
| Eating rate (g/min): Timepoint (min): Test | 0.43 | 0.975 | 0.30 | 0.997 | 0.28 | 0.998 | 0.71 | 0.789 |

**Table S6** Post-hoc linear mixed-effects model estimates for associations between eating rate (ER) and hormone (insulin, C-peptide, glucagon) responses at individual timepoints.

|  | Insulin | | | C-peptide | | Glucagon | | |
| --- | --- | --- | --- | --- | --- | --- | --- | --- |
|  | Estimate | Pr(>\|t\|) | Estimate | | Pr(>\|t\|) | | Estimate | Pr(>\|t\|) |
| ER: Timepoint10 | -2.09E-03 | **0.00824** | -7.94E-04 | | 0.136599 | | 3.03E-04 | 0.607887 |
| ER: Timepoint15 | -1.81E-03 | **0.0219** | -6.90E-04 | | 0.195697 | | 9.46E-05 | 0.87269 |
| ER: Timepoint30 | -6.34E-04 | 0.42177 | -3.87E-04 | | 0.468564 | | 5.38E-04 | 0.363967 |
| ER: Timepoint45 | -9.68E-05 | 0.90244 | -1.15E-04 | | 0.829512 | | -4.11E-04 | 0.487651 |
| ER: Timepoint60 | 2.68E-04 | 0.73416 | 1.71E-04 | | 0.748975 | | 1.11E-04 | 0.850711 |
| ER: Timepoint90 | -8.35E-04 | 0.29011 | -2.07E-04 | | 0.698801 | | 1.55E-03 | **0.009016** |
| ER: Timepoint120 | -2.28E-03 | **0.00398** | -1.40E-03 | | **0.008813** | | 1.03E-03 | 0.083758 |
| ER: Timepoint180 | -2.29E-03 | **0.00379** | -1.81E-03 | | **0.000725** | | 2.65E-05 | 0.964265 |
